# Diagnostic test accuracy of the Emergency Severity Index: a systematic review and meta-analysis

**DOI:** 10.1097/MEJ.0000000000001262

**Published:** 2025-07-18

**Authors:** Bettina Wandl, Jan D. Kellerer, Verena Fuhrmann, Karina Tapinova, Dominik Roth, Gerhard Müller

**Affiliations:** a Department of Nursing Science and Gerontology, Institute of Nursing Science, UMIT TIROL – Private University for Health Sciences and Health Technology, Tyrol; b Department of Emergency Medicine, Medical University of Vienna, Wien, Austria

**Keywords:** diagnostic test accuracy, emergency department, Emergency Severity Index, triage

## Abstract

**Background and importance:**

Efficient triage of emergency patients is crucial for the immediate identification of critically ill individuals and enables rapid interventions to improve patient outcomes.

**Design:**

Systematic review and meta-analysis of the diagnostic test accuracy (DTA) of the Emergency Severity Index (ESI) for identifying critically ill adult patients in the emergency department (ED).

**Settings and participants:**

We considered all studies (case-control and cohort studies) that evaluated the DTA of the ESI in adult patients attending an ED. The outcome of a triage system is the high urgency of treatment, commonly used reference standards are short-term mortality or admission to an ICU.

**Methods:**

We searched four bibliographic databases up to 13 February 2025. Screening, inclusion, data extraction, and assessment of methodological quality followed standard Cochrane methodology. We calculated measures of DTA for all studies against the reference standards and calculated pooled estimates using a bivariate random effects model.

**Main results:**

We included 27 studies, representing 510 777 patients. Methodological quality according to the QUADAS-2 tool was high, except for risk of bias in patient selection, which was high for 12 (44%) studies. A total of 18 studies provided data for the reference standard short-term mortality, with an estimated pooled sensitivity of 81.8 [95% confidence interval (CI): 71.8–88.9], specificity of 70.5 (60.5–78.8), diagnostic odds ratio (DOR) of 10.8 (5.4–21.4), positive likelihood ratio of 2.77 (2.02–3.81), and negative likelihood ratio of 0.26 (0.16–0.41). For the reference standard ICU admission, based on 10 studies, pooled estimates were sensitivity of 81.5 (65.2–91.2), specificity of 81.7 (71.9–88.6), DOR of 19.7 (5.5–70.7), positive likelihood ratio of 4.45 (2.58–7.84), and negative likelihood ratio of 0.23 (0.11–0.49). Those results remained stable in the sensitivity analysis.

**Conclusion:**

ESI showed a moderate-to-high diagnostic accuracy for identifying critically ill patients at the ED. These findings support the role of the ESI guided by a principal understanding of the limitations inherent to any triage tool.

## Introduction

The triage of emergency patients plays a significant role in the quick identification of critically ill patients, which helps to enable rapid interventions with the aim of changing the outcome [1]. Ensuring a smooth flow of patients in emergency departments (EDs) is important to prevent overcrowding and make sure operations remain efficient [2,3]. For this reason, it is essential to optimise the triage processes, improving patient flow and reinforcing emergency care systems to meet the increasing demands of the modern healthcare system.

Köster *et al*. [4] explain this increase as a result of demographic change and the fact that many patients visit EDs themselves instead of seeking treatment from their general practitioner, as a further reason for the increasing number of ED visits. The reason for this is that many patients see EDs as the first point of contact for physical complaints [5]. In addition, many patients decide to visit hospital EDs because of the unavailability of general practitioners [6]. Scherer *et al*. [6] also write that a large number of patients use EDs even though they have a low urgency of treatment.

At the same time, the number of nursing staff, physicians, and equipment in EDs has not increased in equal measure despite the strong increase in treatments in EDs [7]. This described overcrowding of EDs represents a significant problem for patient safety [8]. In a retrospective cohort study, Richardson [9] reported that the risk of 10-day inpatient mortality was 34% higher for patients admitted to the hospital via the ED during overcrowding periods than for patients admitted during nonovercrowding periods.

For this reason, it is necessary to recognise critically ill patients, which represent around 10% of patients in EDs [10], as early as possible to be able to initiate immediate and adequate treatment [11,12]. To be able to assess the severity of illnesses and recognise critically ill patients as early as possible, structured initial assessment systems, also known as triage systems, are used in EDs [13,14]. In addition to its use in military medicine and in the prehospital management of patients injured in major emergencies, clinical triage has also been developed and used in EDs since the mid-20^th^ century [11]. Clinical triage is a risk management system and is intended to control patient flow through the use of standardised and reproducible instruments [15]. With the help of these triage systems, it is possible to systematically categorise emergency patients according to their level of urgency within a few minutes of their arrival and assign them to the correct area of treatment [11]. Implementing a new triage system in a high-volume ED might, however, present several challenges. Currently, there are various triage systems used for the management of patients, and they are divided into three- and five-stage initial assessment tools. According to Christ *et al*. [10], five-step triage systems have better validity and reliability than three-step triage systems and should therefore be used primarily. The most widely used five-stage triage systems include the Australian Triage Scale, the Canadian Triage Scale, the Manchester Triage System, and the Emergency Severity Index (ESI) [10]. Because of increasing treatment numbers and the reduction of resources in EDs [7], the ESI is becoming an increasingly important tool because of the integration of resource requirements [16]. That increase in the use of the ESI has, however, not been without criticism, with reports of potentially high rates of mistriage compared with retrospective chart review [17], inconsistency in assessment of training cases among centres [18], and differences in outcomes compared with prehospital mass casualty triage [19].

This review aims to comprehensively evaluate the diagnostic test accuracy (DTA) of the ESI in detecting high urgency of treatment in adult patients in the ED.

## Methods

We conducted a systematic review using a broad search strategy to identify all studies that evaluated the DTA of the ESI in emergency care with regard to the identification of patients with an increased urgency of treatment or the defined target conditions.

The protocol was registered with the International Prospective Register of Systematic Reviews (PROSPERO CRD42023456720). Methodology followed the Cochrane Handbook for Systematic Reviews of Diagnostic Test Accuracy Version 1.0.0 [20].

### Criteria for considering studies for this review

All studies on the DTA (case-control studies or cohort studies) of the ESI were considered. We excluded all publications of case reports, randomised controlled trials, and all publications that are to be regarded as interventions rather than DTA studies. Included were adults of all sexes presenting at an ED, both by emergency medical services and by any other means of transportation. Prehospital initial assessments and triage in catastrophic cases were excluded.

### Search strategy, information sources, and study selection

The search was unlimited on the year of publication is current as of 13 February 2025.

The following electronic databases were searched to identify relevant studies:

MEDLINE via PubMed, Web of Science Core Collection, SCOPUS, and Embase via Ovid SP (Search strategy, Supplemental digital content 1, https://links.lww.com/EJEM/A503).

The search strategy was developed by B.W. and a health sciences librarian. The final search string was checked using the Peer Review of Electronic Search Strategies: 2015 Guideline Statement [21].

**Fig. 1:**
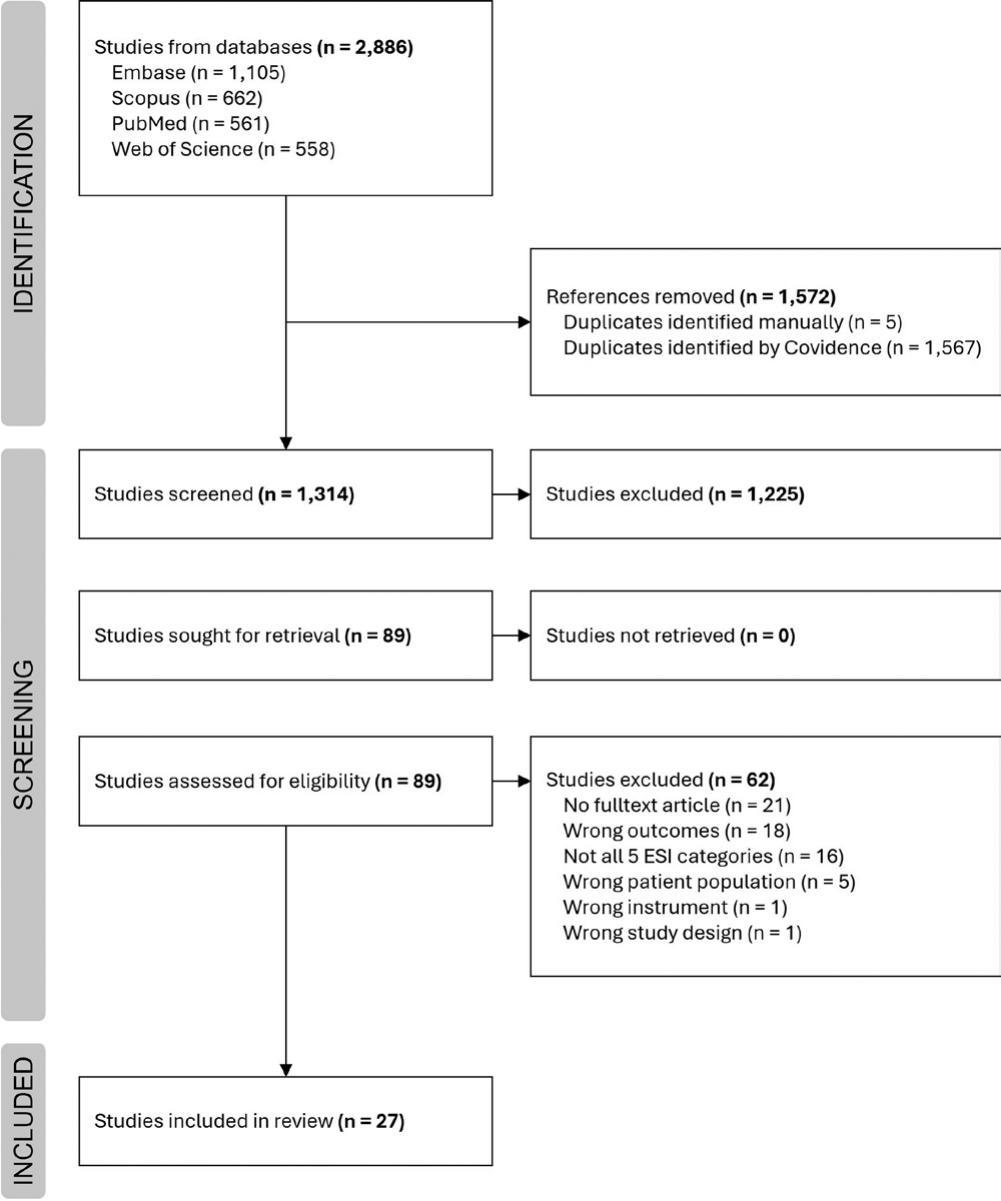
Flow chart of selection progress.

The identified studies were managed with the help of Covidence, a web-based software platform. Duplicates were removed by Covidence and checked manually. B.W. and J.D.K. independently, and in duplicate, screened studies by the title and abstract and based on the previously defined criteria. Studies included after screening were read in full text and selected by B.W. and V.F. in duplicate and independently based on the defined criteria. Differences of opinion were resolved through discussions or through the involvement of G.M. or D.R. as arbitrators. The entire identification and selection phase was graphically documented using a flow chart (Fig. 1). B.W. and K.T. performed the data extraction of the included studies independently and in duplicate, also using Covidence. The template for data extraction was based on the Standards for Reporting of Diagnostic Accuracy [22]. Disagreements were resolved through discussion or by involving D.R. as an arbitrator.

### Quality assessment

The methodological quality was assessed using all four domains of the QUADAS-2 instrument [23], a revision of the original QUADAS instrument [24]. The QUADAS-2 instrument evaluates four key areas: patient selection, index test, reference standard, and process and timing. The first three domains (patient selection, index test, and reference standard) are assessed for both risk of bias and terms of applicability, while the fourth domain, flow and timing, is only assessed for risk of bias [23]. Quality assessment followed the same approach by two independent reviewers as outlined for the search and data extraction.

### Study outcomes

The outcome of interest of a triage system is high urgency of treatment. Accordingly, triage systems aim to identify (‘diagnose’) those at highly urgent need of treatment at the time of triage. There is, however, no generally accepted reference standard for this outcome. For this reason, many studies use admission to an ICU and mortality within a short period of time after triage as a replacement (or ‘reference standard’) for this outcome, and this review follows this practice. For the latter, we used the shortest reported timeframe within each study (for information on timeframe per study, see Supplemental digital content 2, https://links.lww.com/EJEM/A504) [25].

### Data analysis

The ESI was used as the index test. This test is divided into five categories for clinical purposes (with ESI 1 representing the highest, and ESI 5 representing the lowest priority). For clinical purposes, according to the ESI handbook, those categories include a dichotomisation into high-urgency patients with a maximum time to be seen (immediately for ESI 1, 10 min for ESI 2), and lower-urgency patients without any defined timeframe (ESI 3–5). Studies commonly follow this dichotomisation into ESI 1 and 2 vs. 3–5, although sometimes a classification into ESI 1 vs. other is used. In this review, the classification according to the definition of the respective included study was used.

Methods of analysis followed those outlined by Takwoingi [26], and the published protocol [27].

We performed analyses separately for each of the two aforementioned reference standards. First, we calculated two by two tables of urgent vs. nonurgent ESI classification against the reference standard for each individual study. We calculated measures of diagnostic accuracy with 95% confidence intervals (CIs) and presented the results as forest plots. We then performed a bivariate meta-analysis of sensitivity and specificity using a generalised linear mixed-effects model approach using the Stata ‘metandi’-package. We report the pooled (logit-transformed bivariate estimates of) sensitivity and specificity with 95% CIs. We graphed each study’s sensitivity and specificity estimates, summary receiver operating characteristic (sROC) point, and the 95% confidence region around the sROC point to produce a specificity (*x*-axis) vs. sensitivity (*y*-axis) plot. Finally, we performed sensitivity analyses by repeating all calculations while excluding studies representing only specific subpopulations of patients, such as those with active cancer. We did not assess publication bias, as the usefulness of this in reviews of DTA is being discussed controversially [28].

We used RevMan 5.4.1 (The Nordic Cochrane Centre, Copenhagen, Denmark) and StataMP17 (Stata Corp, College Station, Texas, USA) for all analyses.

## Results

### Results of the search

The electronic search identified a total of 2886 studies, with 1314 remaining after deduplication. A total of 1225 studies were excluded during title and abstract screening, and the remaining 89 full texts were screened. Of those, 62 studies were excluded. The reasons for exclusion and the process of selecting the included studies are illustrated in Fig. 1. Finally, a total of 27 studies met inclusion criteria and were included in the systematic review [29–55], 22 (89%) of which were cohort studies.

### Study characteristics

The majority of studies were conducted in academic (tertiary care) hospitals (*n* = 24; 89%), and more than half of the studies were conducted in Europe (*n* = 10, 37%) and Asia (*n* = 11, 41%). Twenty-two studies (82%) were single-centre studies, the median number of participants per study was 1830, with an interquartile range from 565 to 7569. Of the included studies, nine (33%) dealt with a specific subpopulation (patients with active cancer, *n* = 1 [29]; patients with abdominal and chest pain, *n* = 2 [30,54]; geriatric patients, *n* = 4 [31,36,38,49]; and patients with sepsis, *n* = 2 [42,51]), that was addressed in a sensitivity analysis. A complete overview of the selected studies is presented in the characteristics of included studies table (Supplemental digital content 2, https://links.lww.com/EJEM/A504).

### Methodological quality of included studies

**Fig. 2:**
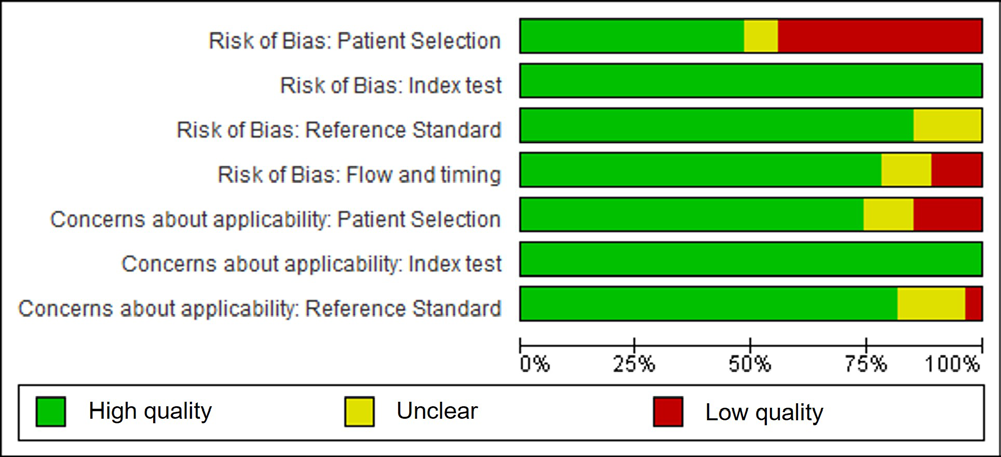
Methodological quality summary: review authors’ judgements about each methodological quality item for each included study.

**Fig. 3:**
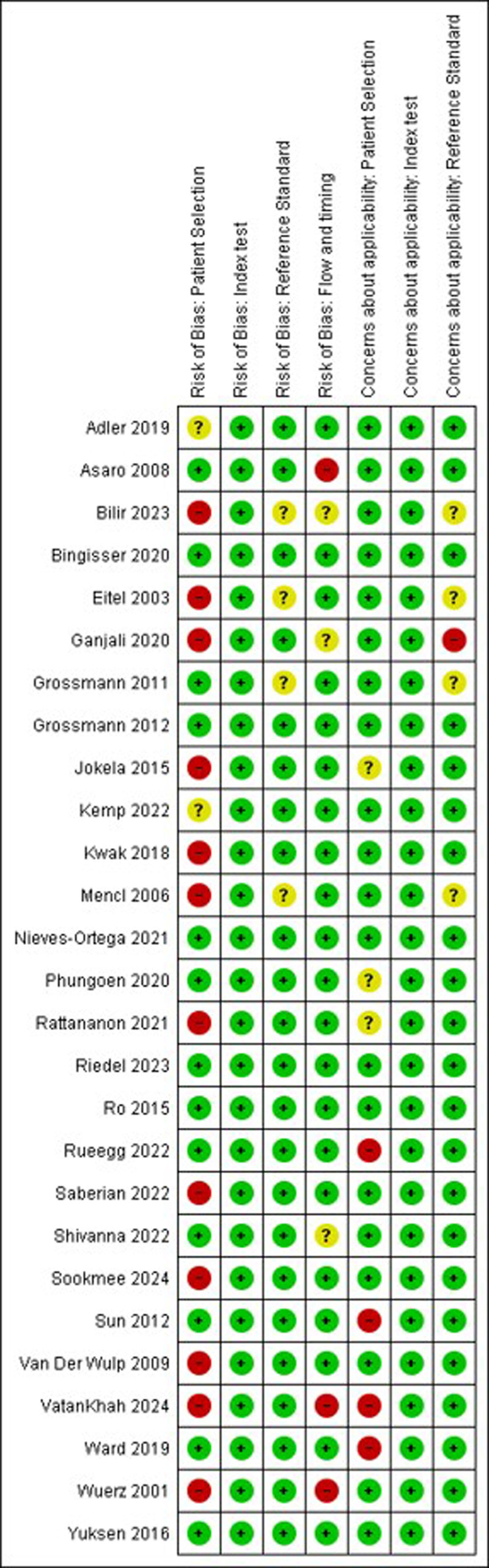
Methodological quality graph: review authors’ judgements about each methodological quality item presented as percentages across all included studies.

Eleven studies (41%) had a high risk of bias in at least one domain [30,31,33,34,37,39,40,43,46,47,49–51,55], two studies [52,54] had a high risk of bias in two domains (Fig. 2). The most common reasons for concern were the type of patient selection, the inclusion and exclusion criteria and the type of sampling and was considered high risk of potential bias in 12 studies (44%) (Fig. 3). In five studies (19%), there were concerns regarding applicability [34,46,49,51,54], mainly in the domain ‘patient selection’.

### Meta-analysis of all included studies

**Fig. 4:**
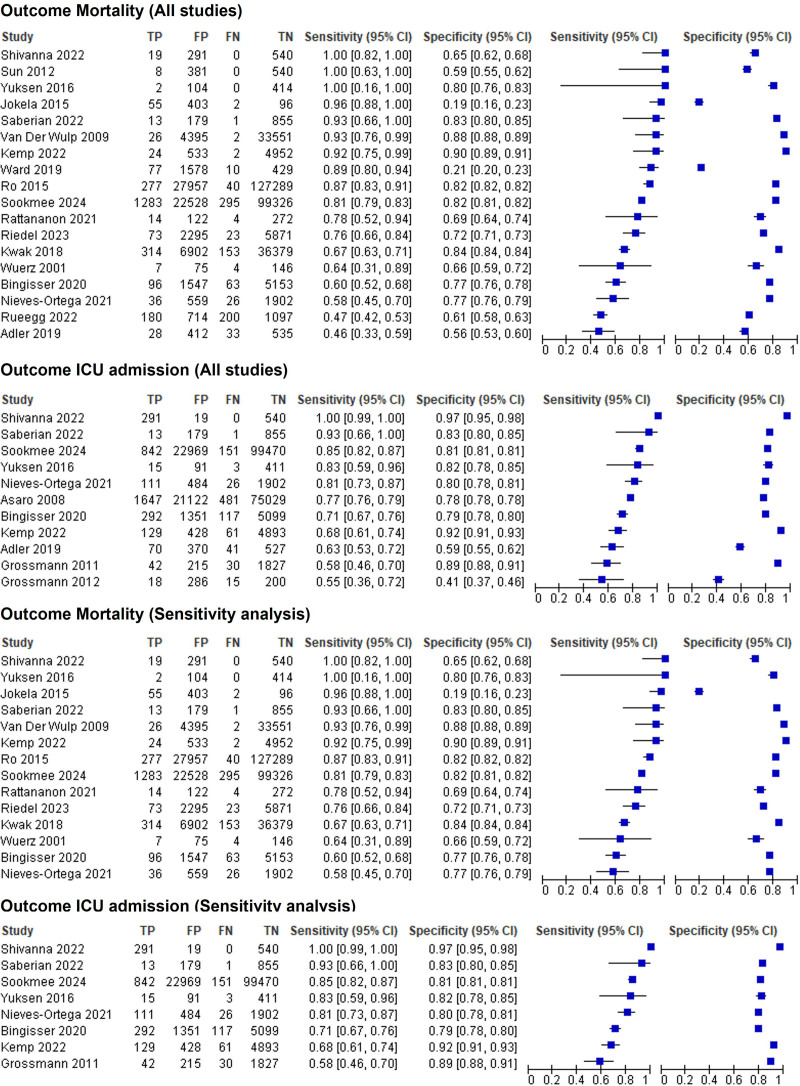
Forest plot, sorted by descending sensitivity. CI, confidence interval; FN, false negative; FP, false positive; TN, true negative; TP, true positive.

Both sensitivity (1.00–0.46 for mortality and 1.00–0.55 for ICU admission) and specificity (0.90–0.19 for mortality, 0.97–0.41 for ICU admission) showed varying results among studies (see Fig. 4 for details).

A total of 18 studies provided sufficient data for meta-analysis for the reference standard of short-term mortality. Meta-analyses resulted in an estimated pooled sensitivity of 81.8 (95% CI: 71.8–88.9), specificity of 70.5 (95% CI: 60.5–78.8), diagnostic odds ratio (DOR) of 10.8 (95% CI: 5.4–21.4), sROC area under curve (AUC) 0.83 (95% CI: 0.80–0.86), positive likelihood ratio of 2.77 (95% CI: 2.02–3.81), and negative likelihood ratio of 0.26 (95% CI: 0.16–0.41).

**Fig. 5:**
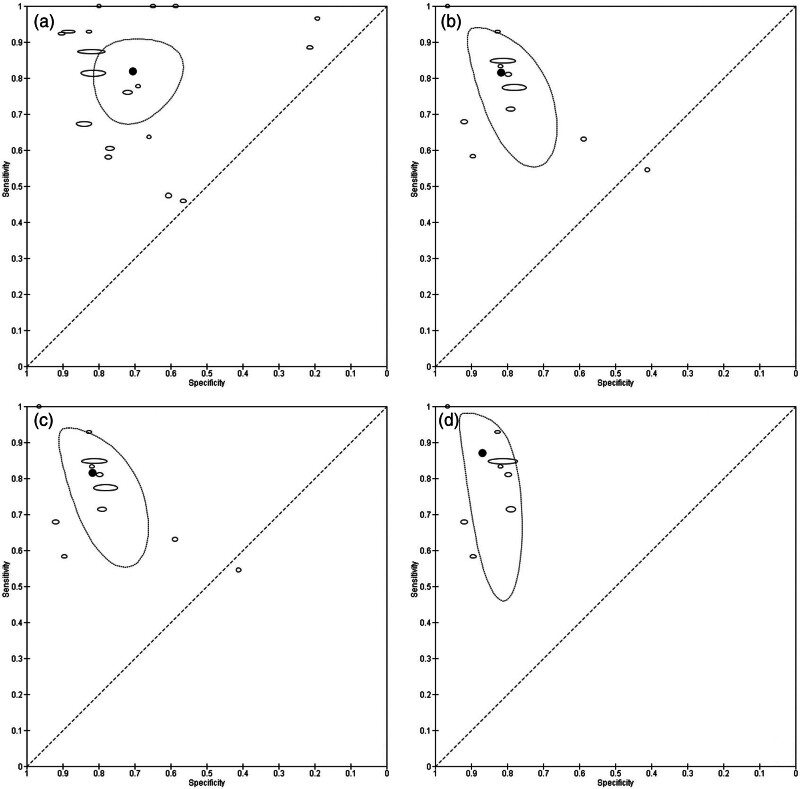
Summary receiver operating characteristic plot. Ellipses represent individual studies, weighted by inverse standard error. The black dot represents the summary point with 95% confidence region (dotted circle). (a) Outcome mortality (all studies). (b) Outcome ICU admission (all studies). (c) Outcome mortality (sensitivity analysis). (d) Outcome ICU admission (sensitivity analysis).

For the reference standard admission to the ICU, a meta-analysis of 11 studies was possible. This resulted in an estimated pooled sensitivity of 81.5 (95% CI: 65.2–91.2), specificity of 81.7 (95% CI: 71.9–88.6), DOR of 19.7 (95% CI: 5.5–70.7), sROC AUC of 0.88 (95% CI: 0.85–0.91), positive likelihood ratio of 4.45 (95% CI: 2.58–7.84), and negative likelihood ratio of 0.23 (95% CI: 0.11–0.49) (See Fig. 5 for a sROC plot).

### Sensitivity analysis

As eight studies included only patients of specific subpopulations (such as patients with active cancer; see section Study characteristics), we performed a sensitivity analysis excluding studies with those special populations. This left 14 studies for meta-analysis for the reference standard of mortality. We found only very minor changes of estimates, all within the CIs of the primary analysis: sensitivity of 83.4 (95% CI: 74.0–89.9), specificity of 75.8 (95% CI: 66.8–83.0), DOR of 15.7 (95% CI: 8.4–29.3), sROC AUC of 0.87 (95% CI: 0.83–0.89), positive likelihood ratio of 3.44 (95% CI: 2.49–4.76), and negative likelihood ratio of 0.22 (95% CI: 0.14–0.35). Results were similar for the reference standard of ICU admissions, although CIs were considerably wider, with only eight studies included: Sensitivity of 87.0 (95% CI: 65.5–96.0), specificity of 86.9 (95% CI: 80.8–91.2), DOR of 44.3 (95% CI: 9.2–213.4), sROC AUC of 0.92 (95% CI: 0.89–0.94), positive likelihood ratio of 6.6 (95% CI: 4.08–11.01), and negative likelihood ratio of 0.15 (95% CI: 0.05–0.47).

## Discussion

We found a substantial number of studies on the diagnostic utility of the ESI. Risk of bias was low for most studies for most domains, with the notable exception of patient selection, which was assessed to be at high risk of bias for 41% of the studies. Excluding those studies had however no impact on pooled estimates.

Estimates for pooled sensitivity were around 80% for both commonly used reference standards (short-term mortality and ICU admission). This implies that although the vast majority of patients in a critical state can be identified at the stage of triage, around a fifth of them are still not being recognised. Although two-thirds of studies showed a sensitivity of over 75%, there was relevant heterogeneity in results, and sensitivity was around or even below 50% for either mortality or ICU admission in a total of five studies [29,35,36,41,46]. Results for specificity were similar [37,51]. All but two of those studies [37,41] covered only special populations (geriatric, cancer, and sepsis) and were excluded from the sensitivity analysis of studies with a general population. The effect of those outlying studies on overall estimates was only modest, however, as demonstrated by the very similar results of the main analysis and the sensitivity analysis.

For an ED-triage tool, identification of low-risk patients could be seen as almost as important as identification of high-risk patients, as this allows a better utilisation of resources for those most in need. Specificity of the ESI was similar to sensitivity when compared with the reference standard of ICU admission, but worse (ca. 70%) for the reference standard of mortality.

Those findings remained virtually unchanged when excluding studies with special populations, such as patients with cancer.

Our findings are mostly consistent with previous publications, although individual studies tend to be overly optimistic, as is often the case. Tanabe *et al*. [56] found that the ESI effectively stratified patients based on the need for urgent resources, aligning with our findings on its high sensitivity and specificity for critical outcomes like mortality and ICU admissions. Another study by McHugh *et al*. [57] confirmed the ESI’s utility in identifying patients who required hospitalisation, which correlates with our findings on ICU admissions. Regarding special patient populations and circumstances, Javidi *et al*. [58] in their study of patients with trauma support the reliability of the ESI in both older and younger patients, although this might have been different during the coronavirus disease 2019 pandemic [59].

### Clinical implications and further directions

Interpretation of the diagnostic performance of the ESI has to take into account the intended role of this tool. A triage system is neither expected to fully diagnose all potentially threatening situations, nor is it used as a final basis for discharge of low-risk patients. Instead, it serves to prioritise huge numbers of patients within a very short period of time, based on rather limited information. Given this intended use, the ESI seems to perform reasonably well. This is underlined by recommendations such as the joint task force of the American College of Emergency Physicians and the Emergency Nurses Association. On the basis of the expert consensus and a review of the available evidence, the task force supported the adoption of a reliable five-level triage scale, stating that either the Canadian Triage and Acuity Scale or the ESI is are good choices for ED triage [60]. Despite those recommendations and the results of our review, healthcare providers need to make sure that the other parts of the emergency screening, diagnosis, and treatment process complement initial triage. If only information from the ESI were used to classify patients as potentially high-risk, one in five of those patients would be overlooked, clearly an unacceptable rate. Those who take care of patients in the ED after the triage process, such as physicians and nurse practitioners, need to be aware of this and make sure that they use proper caution for all patients, including those classified as low risk in the triage process. Similarly, patients initially classified as high risk might easily be relegated to a lower urgency category after performing simple investigations, which were not part of the triage process (such as an ECG).

Our findings also seem to imply similar performance for both general and specific patient populations, such as those with active cancer. This was further underlined by the fact that despite relevant risk for selection bias in a number of studies, this did not have an impact on pooled estimates. Further research might reiterate this assessment or establish a niche for population-specific triage tools.

### Strengths and weaknesses of the review

Our study included a large number of studies, providing a robust and comprehensive analysis of the ESI’s diagnostic accuracy. By conducting sensitivity analyses, we were able to assess the impact of special populations on the ESI’s performance. The focus on mortality and ICU admissions addresses critical outcomes in emergency medicine and provides valuable insights for clinical practice.

In the published study protocol, the minimum age of the included patients was defined as 18 years and older. When screening potentially eligible studies, we realised that those studies used heterogeneous definitions of ‘adult’ patients, with many studies using a minimum age of 14 years. Accordingly, we decided to deviate from the published study protocol on this point and to use the individual studies’ definition of adult patients. Similar to most other diagnostic reviews, the lack of generally accepted methods to assess publication bias for this type of study precludes any inference on whether publication bias might have been present based on our results. We also did not perform a specific search for ‘grey literature’, such as conference abstracts.

### Conclusion

ESI showed a moderate to high diagnostic accuracy for identifying critically ill patients at the ED. These findings support the role of the ESI guided by a principal understanding of the limitations inherent to any triage tool.

## Acknowledgements

This research project was funded by the state of Tyrol, Austria (‘Tiroler Nachwuchsforscher*innenförderung’) (Grant No.: F.47908/6-2023).

### Conflicts of interest

There are no conflicts of interest.

## Supplementary Material



## References

[1] Joseph JW Leventhal EL Grossestreuer AV Wong ML Joseph LJ Nathanson LA. Deep-learning approaches to identify critically Ill patients at emergency department triage using limited information. J Am Coll Emerg Physicians Open 2020; 1:773–781. 33145518 10.1002/emp2.12218 PMC7593422

[2] Baier N Geissler A Bech M Bernstein D Cowling TE Jackson T. Emergency and urgent care systems in Australia, Denmark, England, France, Germany and the Netherlands – analyzing organization, payment and reforms. Health Policy 2019; 123:1–10. 30503764 10.1016/j.healthpol.2018.11.001

[3] Völk S Koedel U Horster S Bayer A D’Haese JG Pfister HW Klein M. Patient disposition using the Emergency Severity Index: a retrospective observational study at an interdisciplinary emergency department. BMJ Open 2022; 12:e057684. 10.1136/bmjopen-2021-057684 PMC9109098 35551090

[4] Köster C Wrede S Hermann T Meyer S Willms G Broge B. Ambulante Notfallversorgung, Analyse und Handlungsempfehlungen. AQUA – Institut für angewandte Qualitätsförderung und Forschung im Gesundheitswesen GmbH; 2016.

[5] Mota Guedes H Aparecida Araújo F Pinto Júnior D Amado Martins JC Machado Chianca TC. Outcome assessment of patients classified through the Manchester Triage System in emergency units in Brazil and Portugal. Invest Educ Enferm 2017; 35:174–181. 29767936 10.17533/udea.iee.v35n2a06

[6] Scherer M Lühmann D Kazek A Hansen H Schäfer I. Patienten in notfallambulanzen. Querschnittstudie zur subjektiv empfundenen behandlungsdringlichkeit und zu den motiven, die notfallambulanzen von krankenhäusern aufzusuchen. Dtsch Arztebl 2017; 114:645–652.

[7] Osterloh F. Notfallversorgung: ambulant oder stationär? Dtsch Arztebl 2016; 113:1809–1810.

[8] Carter EJ Pouch SM Larson EL. The relationship between emergency department crowding and patient outcomes: a systematic review. J Nurs Scholarsh 2014; 46:106–115. 24354886 10.1111/jnu.12055 PMC4033834

[9] Richardson DB. Increase in patient mortality at 10 days associated with emergency department overcrowding. Med J Aust 2006; 184:213–216. 16515430 10.5694/j.1326-5377.2006.tb00204.x

[10] Christ M Dodt C Stadelmeyer U Hortmann M Geldner G Wulf H. Professionalisierung der klinischen Notfallmedizin – Gegenwart und Zukunft [Presence and future of emergency medicine in Germany]. Anasthesiol Intensivmed Notfallmed Schmerzther 2010; 45:666–671. 20960371 10.1055/s-0030-1267533

[11] Christ M Bingisser R Nickel CH. Bedeutung der triage in der klinischen notfallmedizin. Dtsch Med Wochenschr 2016; 141:329–335. 26939102 10.1055/s-0041-109126

[12] Kemp K Alakare J Kätkä M Lääperi M Lehtonen L Castrén M. Effect of age adjustment on two triage methods. BMC Emerg Med 2022; 22:52. 35346062 10.1186/s12873-022-00600-0 PMC8961917

[13] Body R Kaide E Kendal S Foex B. Not all suffering is pain: sources of patients’ suffering in the emergency department call for improvements in communication from practitioners. Emerg Med J 2015; 32:15–20. 24366946 10.1136/emermed-2013-202860

[14] Hortmann M Heppner HJ Popp S Lad T Christ M. Reduction of mortality in community-acquired pneumonia after implementing standardized care bundles in the emergency department. Eur J Emerg Med 2014; 21:429–435. 24384619 10.1097/MEJ.0000000000000106

[15] Mackway-Jones K Marsden J Windle J. Ersteinschätzung in der notaufnahme – das manchester-triage-system (Vol. 3). Hans Huber Verlag 2011; 3:1.

[16] Grossmann FF Delport K Keller DI. Emergency Severity Index. Notfall + Rettungsmedizin 2009; 12:290–292.

[17] Sax DR Warton EM Mark DG Vinson DR Kene MV Ballard DW; Kaiser Permanente CREST (Clinical Research on Emergency Services & Treatments) Network. Evaluation of the emergency severity index in US emergency departments for the rate of mistriage. JAMA Netw Open 2023; 6:e233404. 36930151 10.1001/jamanetworkopen.2023.3404 PMC10024207

[18] Mistry B Stewart De Ramirez S Kelen G Schmitz PSK Balhara KS Levin S. Accuracy and reliability of emergency department triage using the Emergency Severity Index: an international multicenter assessment. Ann Emerg Med 2018; 71:581–587.e3. 29174836 10.1016/j.annemergmed.2017.09.036

[19] Wexler BJ Stahlman BA. Triaging patients prior to the arrival of the mass casualty: Emergency Severity Index equivalency to SALT disaster triage. Am J Disaster Med 2022; 17:127–130. 36494883 10.5055/ajdm.2022.0426

[20] Deeks JJ Wisniewski S Davenport C. Guide to the contents of a Cochrane diagnostic test accuracy protocol. The Cochrane Collaboration; 2013.

[21] McGowan J Sampson M Salzwedel DM Cogo E Foerster V Lefebvre C. PRESS peer review of electronic search strategies: 2015 guideline statement. J Clin Epidemiol 2016; 75:40–46. 27005575 10.1016/j.jclinepi.2016.01.021

[22] Bossuyt PM Reitsma JB Bruns DE Gatsonis CA Glasziou PP Irwig L; STARD Group. STARD 2015: an updated list of essential items for reporting diagnostic accuracy studies. BMJ (Clin Res ed.) 2015; 351:h5527. 10.1136/bmj.h5527 PMC4623764 26511519

[23] Whiting PF Rutjes AW Westwood ME Mallett S Deeks JJ Reitsma JB; QUADAS-2 Group. QUADAS-2: a revised tool for the quality assessment of diagnostic accuracy studies. Ann Intern Med 2011; 155:529–536. 22007046 10.7326/0003-4819-155-8-201110180-00009

[24] Whiting P Rutjes AWS Reitsma JB Bossuyt PMM Kleijnen J. The development of QUADAS: a tool for the quality assessment of studies of diagnostic accuracy included in systematic reviews. BMC Med Res Methodol 2003; 3:25. 14606960 10.1186/1471-2288-3-25 PMC305345

[25] Roth D Heidinger B Havel C Herkner H. Different mortality time points in critical care trials: current practice and influence on effect estimates in meta-analyses. Crit Care Med 2016; 44:e737–e741. 26963325 10.1097/CCM.0000000000001631

[26] Takwoingi Y.Meta-analysis of test accuracy studies in Stata: a bivariate model approach. Version 2.01 http://methods.cochrane.org/sdt/. [Accessed 14 March 2025].

[27] Wandl B Roth D Müller G. Protocol of systematic review and meta-analysis of the diagnostic test accuracy of the Emergency Severity Index; 2023. https://www.crd.york.ac.uk/PROSPEROFILES/456720_PROTOCOL_20230911.pdf. [Accessed 14 March 2025]. 10.1097/MEJ.0000000000001262 PMC12382730 40686481

[28] Begg CB. Systematic reviews of diagnostic accuracy studies require study by study examination: first for heterogeneity, and then for sources of heterogeneity. J Clin Epidemiol 2005; 58:865–866. 16085189 10.1016/j.jclinepi.2005.03.006

[29] Adler D Abar B Durham DD Bastani A Bernstein SL Baugh CW. Validation of the Emergency Severity Index (version 4) for the triage of adult emergency department patients with active cancer. J Emerg Med 2019; 57:354–361. 31353265 10.1016/j.jemermed.2019.05.023 PMC7478143

[30] Asaro PV Lewis LM. Effects of a triage process conversion on the triage of high-risk presentations. Acad Emerg Med 2008; 15:916–922. 18785936 10.1111/j.1553-2712.2008.00236.x

[31] Bilir O Yazici MM Ataş I Ersunan G. Clinical profiles of centenarian patients presenting to the emergency department with an acute disease. Meandros Med Dental J 2023; 24:142–147.

[32] Bingisser R Baerlocher SM Kuster T Ortega RN Nickel CH. Physicians’ disease severity ratings are non-inferior to the emergency severity index. J Clin Med 2020; 9:762. 32168931 10.3390/jcm9030762 PMC7141189

[33] Eitel DR Travers DA Rosenau AM Gilboy N Wuerz RC. The Emergency Severity Index triage algorithm version 2 is reliable and valid. Acad Emerg Med 2003; 10:1070–1080. 14525740 10.1111/j.1553-2712.2003.tb00577.x

[34] Ganjali R Golmakani R Ebrahimi M Eslami S Bolvardi E. Accuracy of the emergency department triage system using the emergency severity index for predicting patient outcome; a single center experience. Bull Emerg Trauma 2020; 8:115–120. 32420397 10.30476/BEAT.2020.46452 PMC7211387

[35] Grossmann FF Nickel CH Christ M Schneider K Spirig R Hoeft A. Transporting clinical tools to new settings: cultural adaptation and validation of the emergency severity index in German. Ann Emerg Med 2011; 57:257–264. 20952097 10.1016/j.annemergmed.2010.07.021

[36] Grossmann FF Zumbrunn T Frauchiger A Delport K Bingisser R Nickel CH. At risk of undertriage? Testing the performance and accuracy of the emergency severity index in older emergency department patients. Ann Emerg Med 2012; 60:317–325. 22401951 10.1016/j.annemergmed.2011.12.013

[37] Jokela K Setälä P Virta J Huhtala H Yli-Hankala A Hoppu S. Using a simplified pre-hospital ‘MET’ score to predict in-hospital care and outcomes. Acta Anaesthesiol Scand 2015; 59:505–513. 25736540 10.1111/aas.12499

[38] Kemp K Alakare J Kätkä M Lääperi M Lehtonen L Castrén M. Accuracy of Emergency Severity Index in older adults. Eur J Emerg Med 2022; 29:204–209. 34954725 10.1097/MEJ.0000000000000900 PMC9042339

[39] Kwak H Suh GJ Kim T Kwon WY Kim KS Jung YS. Prognostic performance of Emergency Severity Index (ESI) combined with qSOFA score. Am J Emerg Med 2018; 36:1784–1788. 29472038 10.1016/j.ajem.2018.01.088

[40] Mencl F Elshove-Bolk J van Rijswijck BT Simons M van Vugt AB. Validation of the emergency severity index (ESI) in self-referred patients in a European Emergency Department. Ann Emerg Med 2006; 48:S24–S24. 10.1136/emj.2006.039883 PMC2660021 17351220

[41] Nieves-Ortega R Brabrand M Dutilh G Kellett J Bingisser R Nickel CH. Assessment of patient mobility improves the risk stratification of triage with the Emergency Severity Index: a prospective cohort study. Eur J Emerg Med 2021; 28:456–462. 34149009 10.1097/MEJ.0000000000000845

[42] Phungoen P Khemtong S Apiratwarakul K Ienghong K Kotruchin P. Emergency Severity Index as a predictor of in-hospital mortality in suspected sepsis patients in the emergency department. Am J Emerg Med 2020; 38:1854–1859. 32739856 10.1016/j.ajem.2020.06.005

[43] Rattananon P Yenyuwadee I Dheeradilok T Boonsoong P Thokanit NS Chimdist S. Predictors of mortality among inter-hospital transferred patients in a middle-income country: a retrospective cohort study. Siriraj Med J 2021; 73:312–321.

[44] Riedel HB Espejo T Bingisser R Kellett J Nickel CH. A fast emergency department triage score based on mobility, mental status and oxygen saturation compared with the emergency severity index: a prospective cohort study. QJM 2023; 116:774–780. 37399089 10.1093/qjmed/hcad160 PMC10559338

[45] Ro YS Shin SD Song KJ Cha WC Cho JS. Triage-based resource allocation and clinical treatment protocol on outcome and length of stay in the emergency department. Emerg Med Australas 2015; 27:328–335. 26075591 10.1111/1742-6723.12426

[46] Rueegg M Nissen SK Brabrand M Kaeppeli T Dreher T Carpenter CR. The clinical frailty scale predicts 1-year mortality in emergency department patients aged 65 years and older. Acad Emerg Med 2022; 29:572–580. 35138670 10.1111/acem.14460 PMC9320818

[47] Saberian P Abdollahi A Hasani-Sharamin P Modaber M Karimialavijeh E. Comparing the prehospital NEWS with in-hospital ESI in predicting 30-day severe outcomes in emergency patients. BMC Emerg Med 2022; 22:42. 35287593 10.1186/s12873-022-00598-5 PMC8922925

[48] Shivanna HK Ramesh AC Rangaswamy KMM. Implementation and evaluation of the five-level emergency triage (emergency severity index tool): a hospital-based, prospective, observational study. J Emerg Pract Trauma 2022; 8:43–48.

[49] Sun B Gabayan G Chiu V Yiu S Derose S. Predictive validity of emergency department crowding measures for inpatient mortality. Ann Emerg Med 2012; 60:S72–S73.

[50] Van Der Wulp I Schrijvers AJP Van Stel HF. Predicting admission and mortality with the Emergency Severity Index and the Manchester Triage System: a retrospective observational study. Emerg Med J 2009; 26:506–509. 19546272 10.1136/emj.2008.063768

[51] Ward HH Kiernan EA Deschler CL Murillo SM Karoly EA Macfarlan JE. Clinical and demographic parameters of patients treated using a sepsis protocol. Clin Ther 2019; 41:1020–1028. 31084993 10.1016/j.clinthera.2019.03.016

[52] Wuerz R. Emergency severity index triage category is associated with six-month survival. ESI triage study group. Acad Emerg Med 2001; 8:61–64. 11136151 10.1111/j.1553-2712.2001.tb00554.x

[53] Yuksen C Sawatmongkornkul S Suttabuth S Sawanyawisuth K Sittichanbuncha Y. Emergency severity index compared with 4-level triage at the emergency department of Ramathibodi University Hospital. Asian Biomed 2016; 10:155–161.

[54] VatanKhah M Malekzadeh J Sharifi MD Mirhaghi A. The diagnostic evaluation of the SINEH cardiopulmonary triage scale and the emergency severity index in the emergency department: a comparative study. Emerg Med Int 2024; 2024:3018777. 38558877 10.1155/2024/3018777 PMC10980548

[55] Sookmee W Liabsuetrakul T Tantarattanapong S Wuthisuthimethawee P. Emergency department length of stay and in-hospital mortality of non-traumatic patients in a university hospital. J Health Sci Med Res 2024; 42:20231018.

[56] Tanabe P Gimbel R Yarnold PR Kyriacou DN Adams JG. Reliability and validity of scores on the emergency severity index version 3. Acad Emerg Med 2004; 11:59–65. 14709429 10.1197/j.aem.2003.06.013

[57] McHugh M Tanabe P McClelland M Khare RK. More patients are triaged using the Emergency Severity Index than any other triage acuity system in the United States. Acad Emerg Med 2012; 19:106–109. 22211429 10.1111/j.1553-2712.2011.01240.x

[58] Javidi S Movahedi M Honarmand A Mirafzal A. Emergency severity index triage in Iran: a comparison between age groups in a trauma center. Adv Emerg Nurs J 2023; 45:145–153. 37106500 10.1097/TME.0000000000000456

[59] James MK Okoye A Wahab V Bolton S Lee SW. Emergency Severity Index (ESI) algorithm in trauma patients: the impact of age during the pandemic. Injury 2023; 54:110875. 37349167 10.1016/j.injury.2023.110875

[60] Fernandes CM Tanabe P Gilboy N Johnson LA McNair RS Rosenau AM. Five-level triage: a report from the ACEP/ENA Five-level Triage Task Force. J Emerg Nurs 2005; 31:39–50; quiz 118. 15682128 10.1016/j.jen.2004.11.002

