# Supplementary material for: Diagnostic test accuracy of the Emergency Severity Index: a systematic review and meta-analysis
Source: Eur J Emerg Med. 2025 Jul 18;32(5):325–34. doi: 10.1097/MEJ.0000000000001262 (PMC12382730; doi:10.1097/MEJ.0000000000001262)
Supplement: Supplementary file 1 [file ejem-32-325-s001.docx]

**Supplemental Digital Content 1: Search strategy**

**History and Search Details for MEDLINE via PubMed**

#1 „Emergency Severity Index“ [Title/Abstract] - 531 results

#2 ESI [Title/Abstract] - 40.028 results

#3 emergen* [Title/Abstract] – 617.906 results

#4 triage [Title/Abstract] – 28.364 results

#5 (#2 AND #3 AND #4) - 269 results

#6 (#1 OR #5) - 561 results

**History and Search Details for Web of Science Core Collection**

#1 „Emergency Severity Index“(All Fields) - 555 results

#2 ESI (All Fields) – 68.244 results

#3 emergen* (All Fields) – 1.262.781 results

#4 triage (All Fields) – 32.752 results

#5 (#2 AND #3 AND #4) - 251 results

#6 (#1 OR #5) - 558 results

**History and Search Details for SCOPUS**

#1 „Emergency Severity Index“ TITLE-ABS-KEY - 624 results

#2 ESI TITLE-ABS-KEY – 66.425 results

#3 emergen* TITLE-ABS-KEY – 1.367.223 results

#4 triage TITLE-ABS-KEY – 40.633 results

#5 (#2 AND #3 AND #4) - 330 results

#6 (#1 OR #5) - 662 results

**History und Search Details for EMBASE**

#1 „Emergency Severity Index“ – 1.010 results

#2 ESI – 49.822 results

#3 emergen* - 1.315.470 results

#4 triage – 46.096 results

#5 (#2 AND #3 AND #4) - 518 results

#6 (#1 OR #5) – 1.105 results
