# Supplementary material for: Diagnostic test accuracy of the Emergency Severity Index: a systematic review and meta-analysis
Source: Eur J Emerg Med. 2025 Jul 18;32(5):325–34. doi: 10.1097/MEJ.0000000000001262 (PMC12382730; doi:10.1097/MEJ.0000000000001262)
Supplement: Supplementary file 2 [file ejem-32-325-s002.docx]

**Supplemental Digital Content 2: Characteristics of included studies (n=25)**

| Study | Geographical region | Design | Clinical setting | Participants | Participant characteristics | cut offs | Inclusion in the meta-analysis measures for | | Excluded from sensitivity analysis |
| --- | --- | --- | --- | --- | --- | --- | --- | --- | --- |
|  |  |  |  |  |  |  | Mortality  (FUP-time) | Admission to ICU |  |
| Adler et al.  (2019) [1] | USA,  Multi center | Cohort study | Academic (tertiary care) hospital | N=1,008 | Ongoing anticancer therapy | 1-2 vs 3 vs 4-5 | X  (30d) | X | X |
| Asaro & Lewis  (2008) [2] | USA,  Single center | Cohort study | Academic (tertiary care) hospital | N=98,279 | All ED patients  with abdominal chest pain | 1-2 vs 3-4-5 |  | X | X |
| Bilir et al.  (2023) [3] | Turkey,  Single center | Cohort study | Academic (tertiary care) hospital | N=222 | Patients aged 100 years and older | no cut offs | X  No data | X  No data | X |
| Bingisser et al.  (2020) [4] | Switzerland,  Single center | Cohort study | Academic (tertiary care) hospital | N=6,859 | All ED patients | no cut offs | X  (30d) | X |  |
| Eitel et al.  (2023) [5] | USA,  Multi center | Cohort study | Combination of different settings | N=1,042 | unclear | no cut offs | X  No data |  | X |
| Ganjali et al.  (2020) [6] | Iran,  Single center | Cross-Sectional Study | Academic (tertiary care) hospital | N=400 | All ED patients ≥ 15 years of age | 1-2 vs 3 vs 4-5 | X  No data | X  No data | X |
| Grossmann et al.  (2011) [7] | Switzerland,  Single center | Cohort study | Academic (tertiary care) hospital | N=2,114 | All ED patients | no cut offs |  | X |  |
| Grossmann et al.  (2012) [8] | Switzerland,  Single center | Secondary Analysis of Cohort study (Subgroup analysis) | Academic (tertiary care) hospital | N=519 | Patients age ≥65 years | no cut offs |  | X | X |
| Jokela et al.  (2015) [9] | Finland,  Multi center | Cohort study | Academic (tertiary care) hospital | N=610 | adult patients (>18 years), met by pre-hospital heli emergency medical services (HEMS) team | no cut offs | X  (in-hospital) |  |  |
| Kemp et al.  (2022) [10] | Finland,  Single center | Cohort study | Academic (tertiary care) hospital | N=5,511 | All ED patients, Patient age ≥18years | unclear | X  (3d, 30d) | X |  |
| Kwak et al.  (2018) [11] | Korea,  Single center | Cohort study | Academic (tertiary care) hospital | N=43,789 | All ED patients, Patient age ≥15years | ESI 3 reference variable | X  (in-hospital) | No data for ESI 4 & 5 |  |
| Mencl et al  (2006) [12] | Europe*,  Multi center | Cohort study | Academic (tertiary care) hospital | N=1,830 | All ED patients, self-referred, Patient age ≥14years | no cut offs | No data | No data | X |
| Nieves-Ortega et al. (2021) [13] | Switzerland, Single center | Cohort study | Academic (tertiary care) hospital | N=2,523 | All ED patients | no cut offs | X  (in-hospital, 30d) | X |  |
| Phungoen et al. (2020) [14] | Thailand,  Single center | Cohort study | Academic (tertiary care) hospital | N=12,556 | Patients with suspected sepsis who visited the ED | 1-2 vs 3-4 | No data | No data | X |
| Rattananon et al. (2021) [15] | Thailand,  Single center | Cohort study | Academic (tertiary care) hospital | N=412 | All ED patients, Patient age ≥16years | no cut offs | X  (3d) |  |  |
| Riedel et al.  (2023) [16] | Switzerland, Single center | Cohort study | Academic (tertiary care) hospital | N=8,278 | All ED patients | no cut offs | X  (1d, 7d, 1y) |  |  |
| Ro et al.  (2015) [17] | Korea,  Single center | Before-after study | Academic (tertiary care) hospital | N=155,567 | All ED patients Patient age ≥18years | ESI 1, ESI 2, ESI 3 and ESI 4-5 | X  (in-hospital) |  |  |
| Rueegg et al.  (2022) [18] | Switzerland, Single center | Cohort study | Academic (tertiary care) hospital | N=2,138 | All ED patients age ≥ 65 years | no cut offs | X  (1y) |  | X |
| Saberian et al. (2022) [19] | Iran,  Single center | Cohort study | Academic (tertiary care) hospital | N=1,048 | All adult patients (age ≥ 16 years) brought to the ED by EMS providers | 1-2 vs 3 vs 4-5 | X  (30d) | X |  |
| Shivanna et al. (2022) [20] | India,  Single center | Cohort study | Academic (tertiary care) hospital | N=850 | All ED patients | no cut offs | X  (in-hospital) | X |  |
| Sookmee et al.  (2024) [21] | Thailand,  Single center | Cohor study | Academic (tertiary care hospital) | N=123,356 | All ED patients, aged at least 15 years | no cut-offs | X  (3d, 28d) | X |  |
| Sun et al.  (2012) [22] | USA,  Single center | Cohort study | Academic (tertiary care) hospital | N=929 | All ED patients age ≥ 65 years | no cut offs | X  (in-hospital) |  | X |
| van der Wulp et al. (2009) [23] | The Netherlands, Multi center | Cohort study | Regular (secondary care) hospitals | N=37,974 | All ED patients | no cut offs | X  (in-hospital) |  |  |
| VatanKhah et al.  (2024) [24] | Iran,  Single center | Cohort study | Academic (tertiary care) hospital | N=145 | Patients with chest pain and dyspnea; at least 18 years old. | 1-2 vs 3-4 | No data | No data | X |
| Ward et al.  (2019) [25] | USA,  Multi center | Cohort study | Regular (secondary care) hospitals | N=2,096 | All ED patients Patient age ≥ 18 years with sepsis criteria | no ESI 5 | X  (in-hospital) |  | X |
| Wuerz  (2001) [26] | USA,  Single center | Cohort study | Academic (tertiary care) hospital | N=202 | All ED patients age ≥ 14 years | no ESI 1 | X  (30d, 252d) |  |  |
| Yuksen et al.  (2016) [27] | Thailand,  Single center | Cohort study | Academic (tertiary care) hospital | N=520 | All ED patients | no cut offs | X  (in-hospital) | X |  |

* country where data was collected is unclear; authors are from Norway and The Netherlands
FUP-time: follow-up time
ICU: intensive care unit

[1] Adler D, Abar B, Durham DD, Bastani A, Bernstein SL, Baugh CW, et al. Validation of the Emergency Severity Index (Version 4) for the Triage of Adult Emergency Department Patients With Active Cancer. Journal of Emergency Medicine 2019; 57 (3):354-361.

[2] Asaro PV, Lewis LM. Effects of a triage process conversion on the triage of high-risk presentations. Academic Emergency Medicine 2008; 15 (10):916-922.

[3] Bilir Ö, Yazıcı MM, Ataş İ, Ersunan G. Clinical Profiles of Centenarian Patients Presenting to the Emergency Department with an Acute Disease. Meandros Medical and Dental Journal 2023; 24 (2):142-147.

[4] Bingisser R, Baerlocher SM, Kuster T, Ortega RN, Nickel CH. Physicians’ disease severity ratings are non-inferior to the emergency severity index. Journal of Clinical Medicine 2020; 9 (3).

[5] Eitel DR, Travers DA, Rosenau AM, Gilboy N, Wuerz RC. The Emergency Severity Index triage algorithm version 2 is reliable and valid. Academic Emergency Medicine 2003; 10 (10):1070-1080.

[6] Ganjali R, Golmakani R, Ebrahimi M, Eslami S, Bolvardi E. Accuracy of the emergency department triage system using the emergency severity index for predicting patient outcome; a single center experience. Bulletin of Emergency and Trauma 2020; 8 (2):115-120.

[7] Grossmann F, Nickel C, Christ M, Schneider K, Spirig R, Hoeft A, et al. Transporting Clinical Tools to New Settings: Cultural Adaptation and Validation of the Emergency Severity Index in German. Annals of Emergency Medicine 2011; 57 (3):257-264.

[8] Grossmann FF, Zumbrunn T, Frauchiger A, Delport K, Bingisser R, Nickel CH. At risk of undertriage? Testing the performance and accuracy of the emergency severity index in older emergency department patients. Annals of Emergency Medicine 2012; 60 (3):317-325.

[9] Jokela K, Setälä P, Virta J, Huhtala H, Yli-Hankala A, Hoppu S. Using a simplified pre-hospital 'MET' score to predict in-hospital care and outcomes. Acta Anaesthesiologica Scandinavica 2015; 59 (4):505-513.

[10] Kemp K, Alakare J, Kätkä M, Lääperi M, Lehtonen L, Castrén M. Accuracy of Emergency Severity Index in older adults. European Journal of Emergency Medicine 2022; 29 (3):204-209.

[11] Kwak H, Suh GJ, Kim T, Kwon WY, Kim KS, Jung YS, et al. Prognostic performance of Emergency Severity Index (ESI) combined with qSOFA score. American Journal of Emergency Medicine 2018; 36 (10):1784-1788.

[12] Mencl F, Elshove-Bolk J, van Rijswijck BT, Simons M, van Vugt AB. Validation of the emergency severity index (ESI) in self-referred patients in a European Emergency Department. ANNALS OF EMERGENCY MEDICINE 2006; 48 (4):S24-S24.

[13] Nieves-Ortega R, Brabrand M, Dutilh G, Kellett J, Bingisser R, Nickel CH. Assessment of patient mobility improves the risk stratification of triage with the Emergency Severity Index: A prospective cohort study. European Journal of Emergency Medicine 2021; 28 (6):456-462.

[14] Phungoen P, Khemtong S, Apiratwarakul K, Ienghong K, Kotruchin P. Emergency Severity Index as a predictor of in-hospital mortality in suspected sepsis patients in the emergency department. American Journal of Emergency Medicine 2020; 38 (9):1854-1859.

[15] Rattananon P, Yenyuwadee I, Dheeradilok T, Boonsoong P, Thokanit NS, Chimdist S, et al. Predictors of Mortality among Inter-Hospital Transferred Patients in a Middle-Income Country: a Retrospective Cohort Study. Siriraj Med J 2021; 73 (5):312-321.

[16] Riedel HB, Espejo T, Bingisser R, Kellett J, Nickel CH. A Fast Emergency Department Triage Score based on Mobility, Mental Status and Oxygen Saturation compared with the Emergency Severity Index: A Prospective Cohort Study. QJM : monthly journal of the Association of Physicians 2023; ((Riedel H.B.; Espejo T.; Bingisser R.; Nickel C.H.) Emergency Department, University Hospital Basel, University of Basel, Basel, Switzerland(Kellett J.) Department of Emergency Medicine, University Hospital, Odense, Denmark).

[17] Ro YS, Shin SD, Song KJ, Cha WC, Cho JS. Triage-based resource allocation and clinical treatment protocol on outcome and length of stay in the emergency department. EMA - Emergency Medicine Australasia 2015; 27 (4):328-335.

[18] Rueegg M, Nissen SK, Brabrand M, Kaeppeli T, Dreher T, Carpenter CR, et al. The clinical frailty scale predicts 1-year mortality in emergency department patients aged 65 years and older. Academic Emergency Medicine 2022; 29 (5):572-580.

[19] Saberian P, Abdollahi A, Hasani-Sharamin P, Modaber M, Karimialavijeh E. Comparing the prehospital NEWS with in-hospital ESI in predicting 30-day severe outcomes in emergency patients. BMC Emergency Medicine 2022; 22 (1).

[20] Shivanna HK, Ramesh AC, Rangaswamy KMM. Implementation and evaluation of the five-level emergency triage (emergency severity index tool): A hospital-based, prospective, observational study. J Emerg Pract Trauma 2022; 8 (1):43-48.

[21] Sookmee W, Liabsuetrakul T, Tantarattanapong S, Wuthisuthimethawee P. Emergency Department Length of Stay and in-Hospital Mortality of Non-Traumatic Patients in a University Hospital. J Health Sci Med Res 2024; 42 (3):e20231018.

[22] Sun B, Gabayan G, Chiu V, Yiu S, Derose S. Predictive validity of emergency department crowding measures for inpatient mortality. Annals of Emergency Medicine 2012; 60 (4):S72-S73.

[23] Van Der Wulp I, Schrijvers AJP, Van Stel HF. Predicting admission and mortality with the Emergency Severity Index and the Manchester Triage System: A retrospective observational study. Emergency Medicine Journal 2009; 26 (7):506-509.

[24] VatanKhah M, Malekzadeh J, Sharifi MD, Mirhaghi A. The Diagnostic Evaluation of the SINEH Cardiopulmonary Triage Scale and the Emergency Severity Index in the Emergency Department: A Comparative Study. Emerg Med Int 2024; 2024:3018777.

[25] Ward HH, Kiernan EA, Deschler CL, Murillo SM, Karoly EA, Macfarlan JE, et al. Clinical and Demographic Parameters of Patients Treated Using a Sepsis Protocol. Clinical Therapeutics 2019; 41 (6):1020-1028.

[26] Wuerz R. Emergency severity index triage category is associated with six-month survival. ESI Triage Study Group. Acad Emerg Med 2001; 8 (1):61-64.

[27] Yuksen C, Sawatmongkornkul S, Suttabuth S, Sawanyawisuth K, Sittichanbuncha Y. Emergency severity index compared with 4-level triage at the emergency department of Ramathibodi University Hospital. Asian Biomedicine 2016; 10 (2):155-161.
